# Adult Children’s Monitoring, Knowledge, and Intergenerational Ambivalence

**DOI:** 10.1093/geroni/igab046.2902

**Published:** 2021-12-17

**Authors:** Noriko Toyokawa, Nancy Darling, Teru Toyokawa

**Affiliations:** 1 Southern Oregon University, Ashland, Oregon, United States; 2 Oberlin College and Conservatory, Oberlin, Ohio, United States; 3 California State University San Marcos, San Marcos, California, United States

## Abstract

Monitoring aging parents' daily life is an essential task for adult children to ensure their parents’ health and safety. The current study examined domains of parents' lives that adult children monitored as caregivers. Based on social domain theory (Smetana, 1999), we hypothesized that adult children would monitor parents’ health and safety issues as respecting parents’ autonomy in other issues. The study also examined how adult children’s belief in need for monitoring and their perception of having actual knowledge of their parents’ behaviors and thoughts would relate to the intensity of their intergenerational ambivalence. Adults who had at least one living parent (N=398, Mage=60, SD=7.7, range 45-77) participated in online surveys. Issues of parents' lives that adult children monitored were categorized into four domains by factor analysis: parents' financial safety, health and physical safety, substance use, and plans with other adult children. A series of regression analyses revealed that adult children's sense of need to know about parents' financial safety was associated with lower ambivalence, B=-.60, SE=.18, β=-.23, p=.001, whereas parents’ physical safety was associated with greater ambivalence, B=.42, SE=.19, β =.17, p=.03. Adult children's perception of their knowledge about parents' financial safety was positively associated with their ambivalence, B=.58, SE=.20, β=.22, p=.004, whereas adult children's perception of parents' physical safety was negatively associated with their ambivalence, B=-.42, SE=.21, β=-.14, p=.05. Different meanings of different types of parents' safety issues for adult children as their caregivers and suggestions for future research will be discussed.

